# Optimizing Parameters with FEM Model for 20CrMnTi Laser Shocking

**DOI:** 10.3390/ma16010328

**Published:** 2022-12-29

**Authors:** Jie Sun, Jiayuan Li, Xiuyu Chen, Zhilong Xu, Yuru Lin, Qingshan Jiang, Junying Chen, Yi Li

**Affiliations:** College of Marine Equipment and Mechanical Engineering, Jimei University, Xiamen 361000, China

**Keywords:** laser shock, single-point, finite element, residual stresses, plastic strain, shock parameters

## Abstract

As a new surface treatment technology, laser shock peening (LSP) is a multi-point overlay process of single-point laser shock. In this study, the finite element method (FEM) was used to build a model of single-point laser shock, and the model was verified by experiments. The difference in residual stresses between the experimental and simulated results was less than 20%. Then, the effects of the stress field and deformation of 20CrMnTi with different laser shock parameters were simulated and analyzed. According to the mechanical response of 20CrMnTi to different laser shock parameters, the optimal shocking process parameters for single-point shocking via LSP were determined to be a shock energy of 5 J, a laser pulse width of 20 ns, and an impact number of 5. Lastly, a simulation of multi-point laser shock was performed with the optimal parameters, and the residual compressive stress values of multi-point impacts are close to those of single-point impacts under the same process conditions.

## 1. Introduction

Carburized steel is widely used for critical components subjected to high speeds, heavy loads, and impacts such as gears and sprockets [1,2,3,4,5] because of its excellent hardenability and impact toughness. The mechanical structural parts are subject to cyclic loads during service, which produces cracks on the surface and leads to fatigue fractures [6,7,8,9,10]. Surface-strengthening methods are used to effectively improve the fatigue life of structural parts.

Surface-strengthening methods include mechanical shot peening, water jetting, rolling, and laser shock [11,12,13,14]. Laser shock peening (LSP) is more efficient because of its high pressure and ultra-high strain rate. It causes significant plastic deformation on the surface of the specimen, introduces residual compressive stress, refines the grain, and improves the fatigue life of the treated specimen [15,16,17,18].

In experimental and theoretical studies, Qin et al. [19] researched the effects of LSP on the surface integrity, high-cycle fatigue performance, and very high cycle fatigue performance of 2024-T351 aluminum alloy and discovered that the fatigue life of 2024-T351 aluminum alloy was significantly improved after the laser impact [20]. Prabhakaran et al. [21] analyzed fatigue damage in automotive structural engineering applications and found a significant improvement in mechanical properties and the extension of the life of mechanical components made of high-strength ultrafine bainitic steel after LSP. In a numerical simulation, Luo et al. [22,23] used ABAQUS to construct an LSP model for TB6 titanium alloy and analyzed the stress distribution. Dai et al. [24,25] investigated the microhardness, residual stress, and rolling contact fatigue properties of 316 stainless steel that was LSP-treated. The results show an improved rolling contact fatigue performance due to the high compressive residual stresses induced. With increasing residual compressive stress, the failure mechanism of the rolling contact fatigue surface changed from delamination to microplastic deformation and altered the original wear mechanism of the rolling contact fatigue surface.

20CrMnTi is a typical carburized steel. If the process parameters are appropriate, LSP can be a suitable reinforcement method to improve the performance of 20CrMnTi. This study aimed to find the appropriate process parameters using a model of single-point-impact 20CrMnTi established based on the finite unit method; the rationality of the model was verified through a series of experiments. Then, we systematically studied the mechanical response of 20CrMnTi with different laser impact parameters to derive the optimal combination of process parameters for single-point-impact 20CrMnTi, and multiple-point laser shock was simulated with the optimized parameters. The results will provide a reference for the LSP strengthening of 20CrMnTi components.

## 2. LSP Process and Model of Single-Point Laser Shocking

### 2.1. LSP Process

**Figure 1:**
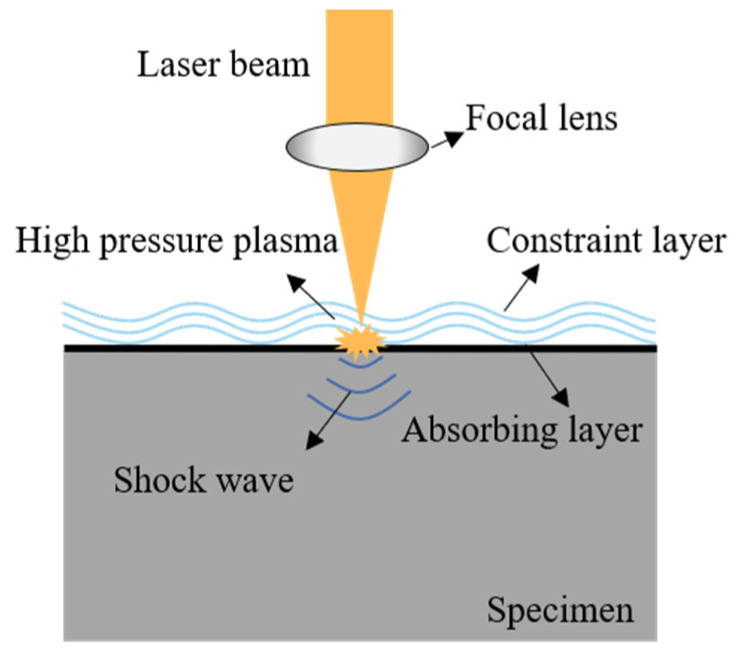
Schematic diagram of laser shock peening (LSP).

LSP utilizes a combination of high peak power density and short pulses of a high-energy laser beam directed at the energy absorption layer above the metal surface. As the energy absorption layer absorbs this energy, instantaneous vaporization occurs, almost simultaneously forming high-temperature and high-pressure plasma shock waves (>1 GPa). These shock waves then propagate inside the specimen under the constraints of the constraint layer. Plastic deformation at an ultra-high strain rate occurs under the action of the shock waves, forming a residual compressive stress layer at a specific depth and amplitude and causing microstructural changes [26,27,28]. A schematic of the LSP process is shown in Figure 1. The laser shock strengthening of parts is a cumulative process of single-point impacts.

### 2.2. Model Construction and Computational Solution

**Figure 2:**
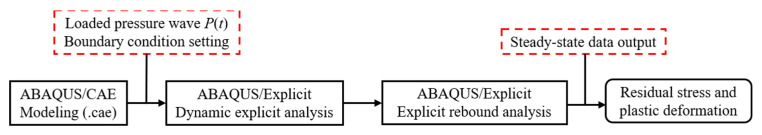
LSP simulation process.

The finite element method was used to construct a numerical simulation model of a 20CrMnTi specimen for single-point shocks. A combination of explicit dynamic analysis and explicit rebound analysis was used to simulate and analyze the equivalent plastic deformation and residual stress distribution of the 20CrMnTi sample subjected to shock waves induced by laser shock. The model was based on the interaction of shock waves with materials, and other phenomena, such as radiation and convection, were not considered of interest to this study. The specific simulation process, outlined in Figure 2, was divided into four main sections: the finite element modeling of the metal specimen, laser shock pressure input, computational solution (including explicit dynamic analysis and explicit rebound analysis), and the analysis of results.

The 20CrMnTi model was an axisymmetric cuboid with a 9 mm × 9 mm × 6 mm size, and an area with a 3 mm diameter was set to be the impact area. The cell mesh was refined in the impact area. 

**Figure 3:**
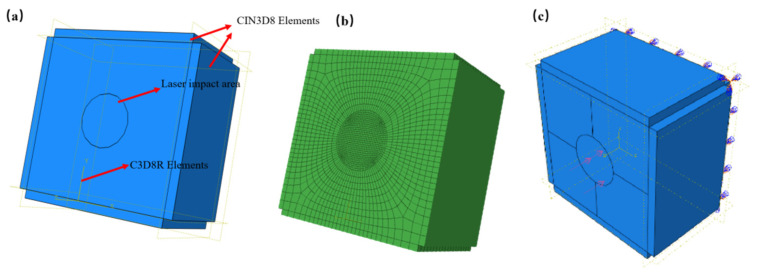
Schematic diagram of the model. (**a**) Partition of model, (**b**) grid distribution, and (**c**) constraints of model.

The neutral-axis algorithm was used to divide the grid, and the central area of the model was the impact spot area and the area of local refinement. The more detailed the meshes, the more accurate the results. The accuracy of residual compressive stress and deformation increased with continuous mesh refinement. When studying the correlation between the mesh size and simulation results, the residual compressive stress and deformation tended to be stable when the mesh size was equal to or smaller than 0.02 mm for the impact area and the mesh size was equal to or smaller than 0.10 mm for other parts of the specimen. Thus, considering the calculation time and solving efficiency comprehensively, the mesh size was 0.02 mm for the impact area and 0.10 mm for other parts of the specimen in the model. In order to ensure that the FE model behaved as closely as possible to reality, infinite boundaries were imposed around the model with a thickness of 0.5 mm, which eliminated the stress reflection, and the bottom of the model was fixed. The element type of infinite boundaries was CIN3D8, while the element type of the other part was C3D8R. The number of elements was 546,240, with an average aspect ratio of 23.5, and the largest aspect ratio was 163.6. The maximum angle of the elements was 149, which was less than the warning value of 160. The average geometric deviation factor was 1.1 × 10^−5^, and the maximum geometric deviation factor was 0.0028. There were no mesh analysis errors. The mesh quality met the analysis requirements, and the calculation results were convergent. The FEM model of the 20CrMnTi specimen is shown in Figure 3. 

A suitable constitutive model is used to describe the response of an object to external action in continuous medium mechanics [29,30]. Constitutive models include the Elastic-Perfectly Plastic (EPP), Zerilli–Armstrong (Z-A), and the widely used Johnson–Cook (J-C) models [31]. The EPP model ignores the strengthening effect of the material. The Z-A and J-C models account for the material’s thermal-softening and strain-hardening components and their response to high-strain-rate loadings [32,33]. The JC material model is extremely sensible for plastic deformation and a high strain rate. Thus, the J-C structure model was adopted in the present study, and stress σ in the JC model is represented by Equation (1).

(1) $$\sigma =\left(A+B\cdot \epsilon^{n}\right)\left(1+C\cdot \ln\frac{\dot{\varepsilon }}{\varepsilon_{0}}\right)\left[1-{\left(\frac{T-T_{1}}{T_{2}-T_{1}}\right)}^{m}\right]$$

where σ denotes the flow stress, ε is the equivalent plastic strain, is the plastic strain rate, ε_0_ is the reference strain rate under quasi-static loading, T is the transition temperature, T1 is the room temperature, and T2 is the material melting point. A represents the yield stress strength under quasi-static loading, B and n are constants reflecting the strain hardening of the material, C is the strain rate constant, and m is the temperature softening coefficient. The equation considers the relationship between the flow stress, strain hardening criterion, high strain rate, and thermal effects. However, in the case of laser shock peening with water confinement, thermal softening can be considered negligible [34]. LSP does not consider thermal effects, and the temperature field can be neglected, which is simplified in Equation (2). In the current study, as LSP with water confinement was numerically modeled, only the A, B, C, and n parameters are considered. The related parameters in the J-C constitutive model of 20CrMnTi are illustrated in Table 1 [1].

(2) $$\sigma =\left(A+B\cdot \varepsilon^{n}\right)\left(1+C\cdot \ln\frac{\dot{\varepsilon }}{\varepsilon_{0}}\right)$$

The shock wave pressure was set in the spot area, the shock solution time was selected based on the energy balance principle, and 4000 ns was selected as the solution time. To ensure the stability of the stress field, a rebound analysis was performed. Unlike the traditional implicit rebound method, we used a multi-analytical step strategy for the explicit rebound based on the kinetic energy tending to 0. In the process of continuously extending the length of the rebound analysis step, it can be found that the kinetic energy decreases continuously until it approaches 0 with the increase in the analysis step time. In a single-point single impact, when the rebound analysis step duration is 96,000 ns, the kinetic energy tends to 0. The rebound time was determined as 96,000 ns.

In the post-processing stage, residual stresses in the vertical and horizontal directions and the equivalent plastic strain (PEEQ) were selected for each node in the horizontal and depth directions according to the characteristics of the stress and strain fields of single-point LSP. 

## 3. Single-Point Laser Shock Experiments

### 3.1. Experimental Conditions

**Figure 4:**
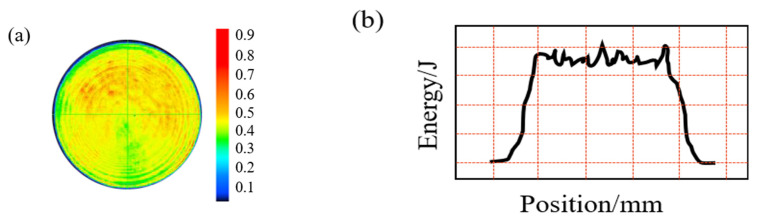
Spatial energy distribution of the flat-top laser beam. (**a**) Spatial energy distribution and (**b**) actual profile.

Experiments were conducted by using the LAMBER-12 solid-state laser, which was made by Zhuo Radium. The laser beam used for LSP is a nanosecond laser. Q-switched Nd made the laser used in the study: a YAG high-power pulsed laser system with a wavelength of 1064 nm. The space energy of the laser has a flat-top distribution, and the beam energy’s actual profile is shown in Figure 4. The intensity of laser beam energy is uniformly distributed approximately in the spot zone.

In the experiments, the 20CrMnTi specimens were shocked by a single laser pulse with the laser shock process parameters outlined in Table 2. The spot diameter was defined as 3 mm based on the structure of the laser impact device, and the laser pulse width was defined as 20 ns. During the experiments, a 2 mm thick flow of deionized water was used as the constraint layer, and 0.1 mm thick black tape was used as the absorption layer.

### 3.2. Detection Methods

The residual stresses in the surface and depth directions of the strengthened area were measured using an HDS-I type X-ray residual stress tester. The residual stresses on the surface were measured at every 0.2 mm spacing. The surface material was removed layer by layer with electrolytically polishing, and then the residual stresses in the depth direction were measured at every 0.05 mm of depth. 

### 3.3. Experimental Results and Analysis

**Figure 5:**
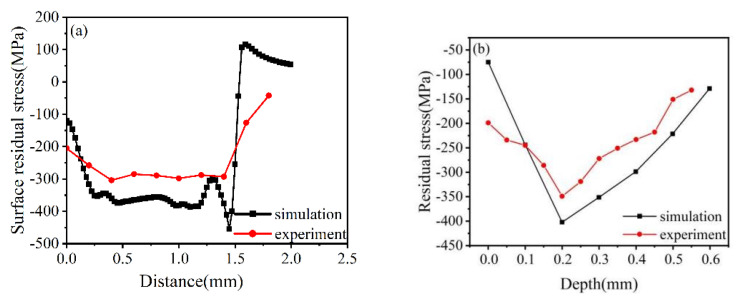
Simulation and experimental residual stress comparison: (**a**) surface orientation; (**b**) depth orientation.

The residual stresses at all measurement points were measured thrice and averaged to reduce the experimental error. The results in the surface and depth directions were compared with the numerical simulation results. As illustrated in Figure 5, the variation trend of the simulated surface residual stress is the same as that of the experimental results, with a difference of about 20%.

At the same time, the variation trend of residual compressive stress along the depth direction obtained from the simulation is the same as that of the experimental results, and the difference between values is about 18%. This meant that the simulation results were in good agreement with the experimental results, the residual stress variation trends were the same, and the finite element model was reasonable.

## 4. Results and Discussion

### 4.1. Mechanical Response of Materials to Different Laser Pulse Energies

**Figure 6:**
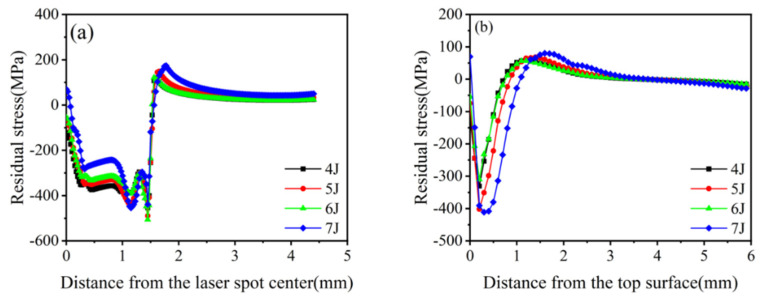
Residual stresses in 20CrMnTi at different laser pulse energies. (**a**) Surface residual stress distribution; (**b**) Depth direction residual stress distribution.

**Figure 7:**
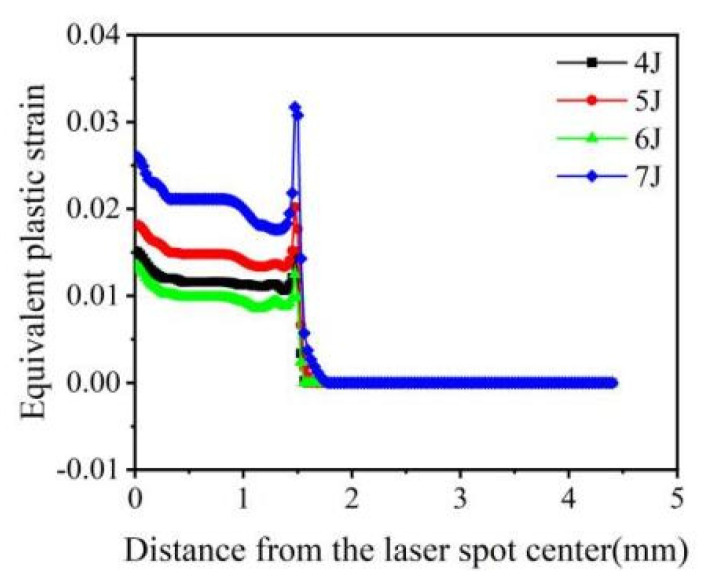
Equivalent plastic strain of 20CrMnTi at different laser pulse energies.

The peak pressure of the shock wave generated by laser shock differed depending on the shock energy, which affected the material’s mechanical response. The pressure waves were set for loading simulations corresponding to laser pulse energies of 4, 5, 6, and 7 J. The positive stress components S11 and PEEQ in the vertical and horizontal directions were extracted (Figure 6 and Figure 7).

The surface residual compressive stress was negatively correlated with the laser pulse energy when the energy increased from 4 to 7 J (Figure 6a). When the energy increases to 7 J, the residual tensile stress appears in the center of the spot, which implies the occurrence of an overshot peening phenomenon, which is not conducive to the improvement in the fatigue strength of the material. The residual compressive stress along the depth direction first increased with the increase in laser pulse energy and then stabilized. As the maximum residual compressive stress was approximately 200 μm from the surface, the residual compressive stress affected approximately 0.9 mm of the layer. The maximum residual compressive stress tended to saturate when the laser pulse energy was 5 J (Figure 6b). The tensile residual stress around the center of the spot was observed, known as the “residual stress hole,” due to the convergence of surface release waves and the interaction between impact and inertial forces on the material surface [35,36]. The tensile residual stress around the edge was due to the “boundary effect.”

The equivalent plastic strain was positively correlated with the change in laser pulse energy (4 J, 5 J, and 7 J) (Figure 7). However, when the pulse energy was 6 J, the equivalent plastic strain was minimum. With the increase in energy, surface hardening becomes more serious. When the energy was 5 J, saturation occurred. When the energy increased to 6 J, the equivalent plastic strain decreased due to the action of a reverse loose wave. When the energy further increased to 7 J, cracks might occur on the surface, surface relaxation would occur, and deformation further increased. The equivalent plastic strain at the center of the light spot was large, which was caused by the reverse plastic deformation of the material caused by the “residual stress hole.” The equivalent plastic strain at the edge of the optical spot sharply changed and became 0. The nature of equivalent plastic strain distribution is highly uneven, as residual stress and tensile residual stress are observed around the center and at the edge of the impact spot. The smaller equivalent plastic strain at the central region of the spot is caused by the reverse plastic deformation of the sparse wave reverse loading [37].

In summary, the optimal laser pulse energy of 20CrMnTi was 5 J.

### 4.2. Mechanical Response of Materials at Different Pulse Widths

**Figure 8:**
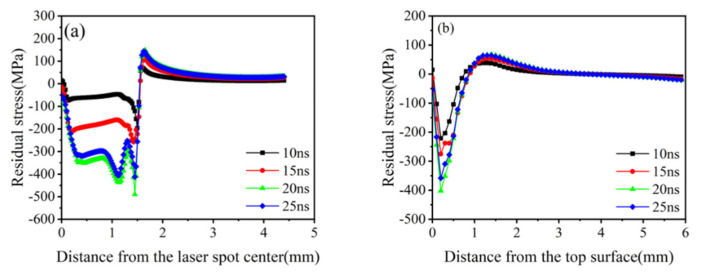
Residual stresses in 20CrMnTi at different pulse widths. (**a**) Surface residual stress distribution; (**b**) Depth direction residual stress distribution.

**Figure 9:**
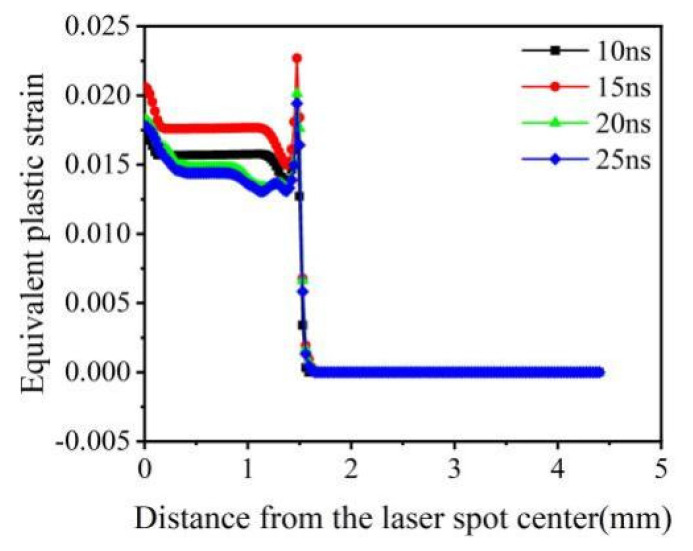
Equivalent plastic strain of 20CrMnTi at different pulse widths.

When the laser pulse energy is constant, different laser pulse widths will lead to different shock wave pressure peaks and action durations. The laser pulse width affects the mechanical response of the material by changing these two parameters. At 5 J energy, the laser pulse widths were selected as 10, 15, 20, and 25 ns, and the corresponding shock wave pressure curves were set for the loading simulation (Figure 8 and Figure 9).

Figure 8a indicates that the surface residual compressive stress increases with the pulse width and saturates at 20 ns. As shown in Figure 8b, a 1 mm residual-compressive-stress-affected layer is formed in the depth direction. The maximum residual compressive stress along the depth direction first increases and then decreases with the increase in the pulse broadband and has a maximum residual compressive stress at 20 ns. Although the pulse width is negatively correlated with the peak shock wave pressure, the duration of the shock wave action also affects the mechanical response of the material. As displayed in Figure 7, there is a competitive relationship between the peak pressure of the shock wave and the duration of action. When the pulse width is less than 20 ns, the material is more sensitive to the impact duration, and the shock duration is at an advantage over the competition. When the pulse width is greater than 20 ns, the material is more sensitive to the pressure peak, and pressure peaks have an edge over the competition.

Figure 9 suggests that the equivalent plastic strain is much less sensitive to the pulse width than the residual stress. The strain gradient at the edge of the spot and the equivalent plastic strain at the center of the spot is large. This phenomenon is caused by the presence of residual stress holes causing reverse plastic deformation, corresponding to the absence of compressive stress at the center of the spot. In summary, the optimal pulse width of the 20CrMnTi laser impact is 20 ns.

### 4.3. Mechanical Responses of Materials to Different Impact Numbers

**Figure 10:**
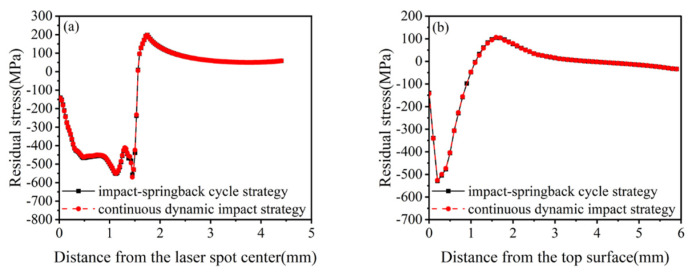
Residual stresses in 20CrMnTi under two impact strategies with double impacts. (**a**) Surface residual stress distribution; (**b**) Depth direction residual stress distribution.

**Figure 11:**
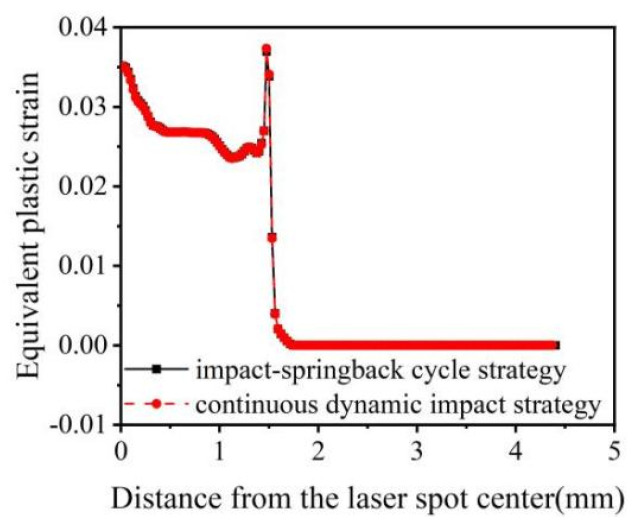
Equivalent plastic strain of 20CrMnTi under two impact strategies with double impacts.

There are two ways to accomplish this step during a simulation with different shock times. First, each impact process consists of two analysis steps: the first constitutes an impact–rebound cycle strategy, and the second is the adoption of a continuous dynamic impact strategy, where multiple impact analysis steps are first set up, and then the last analysis step is used for the rebound analysis. The double-impact simulation was carried out using these two strategies (Figure 10 and Figure 11).

Figure 10 and Figure 11 depict that the stress and strain fields obtained from the above two strategies are the same for double impacts. Still, compared with the impact–rebound cycle strategy, the continuous dynamic impact strategy has a significant advantage in terms of calculation time. With the increase in the number of impacts, this advantage will become more significant; therefore, the continuous dynamic impact strategy was selected for multiple single-point impacts.

**Figure 12:**
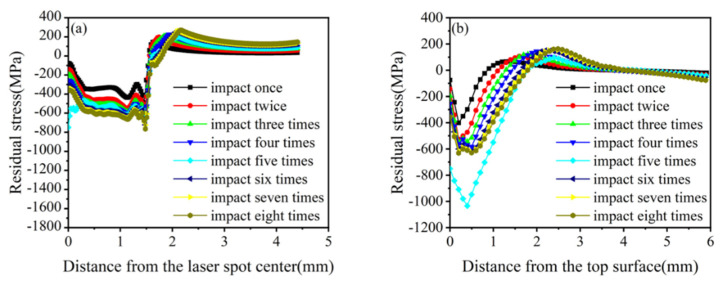
Residual stress of 20CrMnTi for different impact numbers. (**a**) Surface residual stress distribution; (**b**) Depth direction residual stress distribution.

**Figure 13:**
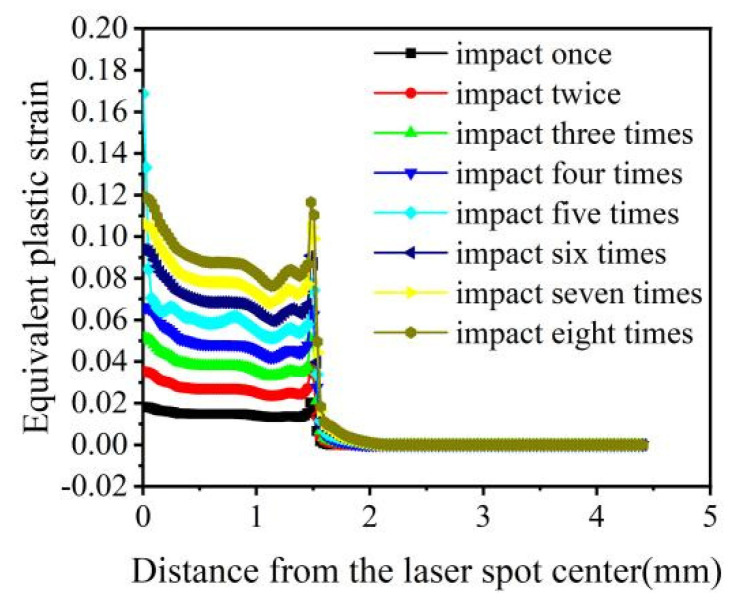
Equivalent plastic strain of 20CrMnTi for different impact numbers.

Unlike the way that the impact energy and pulse width change the mechanical response, changing the number of impacts does not affect the peak pressure of the shock wave or the duration of a single loading, which mainly affects the mechanical response of the material through the superposition of different times of the same load. This study selected a laser with an energy and a pulse width of 5 J and 20 ns, respectively, for 1–8-impact simulations (Figure 12 and Figure 13).

Figure 12a depicts that the surface residual compressive stresses increase when the number of impacts is increased from 1 to 8. However, the residual tensile stress appears in the center of the spot with five impacts. Figure 12b shows that the residual compressive stress along the depth direction increases with the number of impacts when it increases from 1 to 5, reaching a maximum of 5. When the number of impacts increases from 6 to 8, the residual surface stress is also positively correlated with the number of impacts. Still, both are smaller than the residual compressive stress when the number of impacts is five. In addition, except for the case of five shocks, the center of the spot shows a serious “residual stress hole” phenomenon. From the comprehensive surface and depth direction analysis, all five impacts produce optimal stress-strengthening effects; the residual compressive stress affects layer depths greater than 1 mm, and the maximum residual compressive stress is 1085 MPa.

The equivalent plastic strain is positively correlated with the number of impacts, except when the number of impacts is five (Figure 13). When the impact number is five, the equivalent plastic strain suddenly increases and reaches a maximum. The change laws of residual surface stress, surface-layer residual stress, and equivalent plastic deformation are integrated, and the optimal impact parameter of 20CrMnTi is determined to be five times.

### 4.4. Results of Multiple-Point Laser Shock

**Figure 14:**
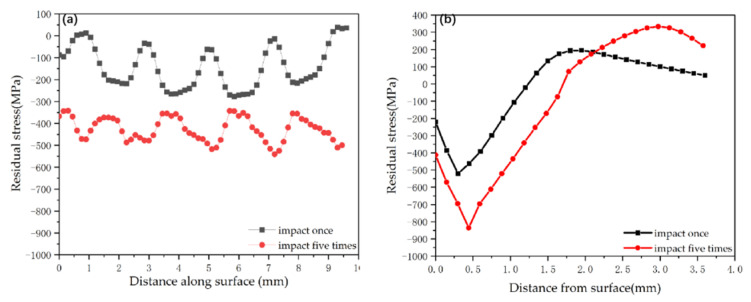
Residual stress of 20CrMnTi of multiple-point laser shock. (**a**) Surface residual stress distribution; (**b**) Depth direction residual stress distribution.

According to the optimal impact parameter combination of single-point impacts obtained above, the multi-point impact was carried out in combination with a 30% lap rate. The results are shown in Figure 14. When the laser pulse energy was selected as 5 J, the pulse width was 20 ns, the number of impacts was 5, the lapping rate was 30%, and the values of residual surface stress and residual stress along the depth direction were obtained. When the impact number was five, the residual surface stress fluctuated around 450 Mpa, and the maximum residual stress along the depth was about 880 Mpa. In contrast, the corresponding values of residual stress were 200 MPa and 510 MPa for one impact. Comparing Figure 13 and Figure 14, as multi-point shocks are the accumulation of single-point shocks, residual compressive stress values of multi-point impacts are close to those of single-point impacts under the same process conditions.

## 5. Conclusions

In this study, a three-dimensional axisymmetric single-point laser impact strengthening model was established using ABAQUS as a platform, and the residual stress distribution in the impact area was compared between simulations and single-point laser impact experiments to verify that the constructed finite element model is reasonable.

1. Using the model to calculate and analyze the effects of the laser impact energy, the laser pulse width, and the number of impacts on the mechanical response of 20CrMnTi, the residual compressive stress on the surface after impact strengthening increases and then decreases as the laser impact energy increases. Further, the maximum residual compressive stress on the surface layer increases and then remains unchanged, with 5 J being the cut-off point.
2. With the increase in the laser pulse width, the surface and surface-layer residual compressive stresses after laser shock peening increase and decrease, reaching their maxima at 20 ns. With the increase in the number of laser shocks, the surface and surface-layer residual compressive stresses after laser shock peening increase and then decrease, reaching maxima at five impacts.
3. A comprehensive analysis shows that the optimal parameters for 20CrMnTi are 5 J, 20 ns, and 5 (impact energy, laser pulse width, and impacts, respectively). In addition, comparing the stress and strain fields between the impact–rebound cycle strategy and ongoing dynamic impact strategy using the same impact parameters, the continuous dynamic impact strategy is selected, saving computing resources, and it was applied in the subsequent multiple-point impact simulations with the optimal parameters.
4. As multi-point shocks are the accumulation of single-point shocks, the residual compressive stress values of multi-point impacts are close to those of single-point impacts under the same process conditions. The results provide a reference for the process selection for the laser shock peening of 20CrMnTi.

## Figures and Tables

**Table 1:** Johnson–Cook parameters of 20CrMnTi.

A	B	n	c
720	712	0.68	0.01

**Table 2:** Laser shock process parameters.

Parameters	Values
Pulse energy/J	4 J
Pulse width/ns	20 ns
Spot diameter/mm	3 mm
Wavelength/nm	1064 nm
